# Comment On: Association of HOTAIR rs1899663 G>T Polymorphism with Colorectal Cancer in the Turkish Population: A Case–Control Study

**DOI:** 10.5152/tjg.2023.23179

**Published:** 2023-07-01

**Authors:** Juan Pablo Meza-Espinoza, Evelia Leal-Ugarte, Verónica Judith Picos-Cárdenas

**Affiliations:** 1 Autonomous University of Tamaulipas Faculty of Medicine and Computer Systems Engineering, Matamoros, Tamaulipas, Mexico; 2 Genetics Laboratory, Autonomous University of Sinaloa Faculty of Medicine, Culiacan, Sinaloa, Mexico

Dear Editor,

We read with great interest the paper entitled “Association of *HOTAIR *rs1899663 G>T Polymorphism with Colorectal Cancer in the Turkish Population: A Case–Control Study” by Yalınbaş et al.^1^ published online in *The Turkish Journal of Gastroenterology*. The article aimed to evaluate the association between the *HOTAIR* gene rs1899663 G>T polymorphism and colorectal cancer (CRC) risk in a Turkish population. The authors stated the G allele had a protective role against CRC in this population but that it would be appropriate to conduct this research with a larger sample to confirm the finding. Undoubtedly, this study is relevant because it is the first to demonstrate an association between CRC and the *HOTAIR* rs1899663 polymorphism in a Caucasian population. However, we would like to make a few observations. 

First, the authors genotyped the patients using DNA extracted from tumor cells. However, genetic association studies should be performed on genomic DNA extracted from non-cancerous cells because cancer cells tend to develop genetic changes related to tumor evolution,^2^ which could bias the results.

Second, the Hardy–Weinberg equilibrium (HWE) test was omitted. We performed this analysis based on the reported genotype frequencies. The control group was in HWE (*χ* ^2^ = 0.2066, *P* = .65), but the CRC sample showed an HWE deviation (*χ* ^2^ = 3.8852, *P* = .049). This can be explained by the overrepresentation of the T allele, and thus the TT genotype, in the patients.

Third, while the conclusion is appropriate, the statistical analysis is not. The authors concluded that there was an association between the T allele and CRC susceptibility, but they overlooked the corresponding odds ratios. Instead, they reported a protective effect for the G allele in Table 2. We respectfully suggest an analysis that we believe is consistent with the conclusion and helps clarify the study by specifying the risk for T allele carriers under different inheritance models. Thus, according to the logistic regression test, the T allele showed a significant association with CRC [G vs. T; OR = 1.69 (1.12-2.55), *P *= .012]; the TT genotype displayed a high CRC susceptibility under a codominant model [GG vs. TT; OR = 4.18 (1.84-9.48), *P* = .0005], but the GT genotype did not [GG vs. GT; OR = 1.52 (0.79-2.92),* P* = .21]. Furthermore, this increased risk was confirmed under the dominant and recessive models [GG vs. GT + TT; OR = 2.13 (1.17-3.89), *P* = .013, and GG + GT vs. TT; OR = 3.32 (1.59-6.94), *P* = .001, respectively].

On the other hand, in this order of ideas, we propose another statistical approach to analyze the association between the polymorphism and the clinicopathological characteristics of the patients, which yields similar results but shows the odds ratios. Thus, in line with the authors’ findings, the only feature that showed a significant risk for the TT genotype was cancer recurrence under a recessive model [GG + GT vs. TT; OR = 9.3 (1.0-88.0), *P* = .04].

We believe that these observations do not discredit this study. Rather, they complement it.
